# *Mycobacterium tuberculosis* senses host Interferon-γ via the membrane protein MmpL10

**DOI:** 10.1038/s42003-022-04265-0

**Published:** 2022-12-01

**Authors:** Mohamed Ahmed, Jared Mackenzie, Liku Tezera, Robert Krause, Barry Truebody, Diana Garay-Baquero, Andres Vallejo, Katya Govender, John Adamson, Hayden Fisher, Jonathan W. Essex, Salah Mansour, Paul Elkington, Adrie J. C. Steyn, Alasdair Leslie

**Affiliations:** 1 Africa Health Research Institute, Durban, 4001 South Africa; 2 College of Health Sciences, School of Laboratory Medicine & Medical Sciences, University of KwaZulu Natal, Durban, 4001 South Africa; 3 NIHR Biomedical Research Centre, School of Clinical and Experimental Sciences, Faculty of Medicine, University of Southampton, Southampton, SO16 6YD UK; 4 Department of Infection and Immunity, University College London, London, WC1E 6BT UK; 5 Biological Sciences, University of Southampton, Southampton, SO17 1BJ UK; 6 Centre for Cancer Immunology, University of Southampton, Southampton, SO16 6YD UK; 7 School of Chemistry, University of Southampton, Southampton, SO17 1BJ UK; 8 Institute for Life Sciences, University of Southampton, Southampton, SO17 1BJ UK; 9 Department of Microbiology, University of Alabama at Birmingham, Birmingham, 35294 USA

**Keywords:** Pathogens, Infectious diseases

## Abstract

*Mycobacterium tuberculosis (Mtb)* is one of the most successful human pathogens. Several cytokines are known to increase virulence of bacterial pathogens, leading us to investigate whether Interferon-γ (IFN-γ), a central regulator of the immune defense against *Mtb*, has a direct effect on the bacteria. We found that recombinant and T-cell derived IFN-γ rapidly induced a dose-dependent increase in the oxygen consumption rate (OCR) of *Mtb*, consistent with increased bacterial respiration. This was not observed in attenuated Bacillus Calmette–Guérin (BCG), and did not occur for other cytokines tested, including TNF-α. IFN-γ binds to the cell surface of intact *Mtb*, but not BCG. Mass spectrometry identified mycobacterial membrane protein large 10 (MmpL10) as the transmembrane binding partner of IFN-γ, supported by molecular modelling studies. IFN-γ binding and the OCR response was absent in *Mtb Δmmpl10* strain and restored by complementation with wildtype *mmpl10*. RNA-sequencing and RT-PCR of *Mtb* exposed to IFN-γ revealed a distinct transcriptional profile, including genes involved in virulence. In a 3D granuloma model, IFN-γ promoted *Mtb* growth, which was lost in the *Mtb Δmmpl10* strain and restored by complementation, supporting the involvement of MmpL10 in the response to IFN-γ. Finally, IFN-γ addition resulted in sterilization of *Mtb* cultures treated with isoniazid, indicating clearance of phenotypically resistant bacteria that persist in the presence of drug alone. Together our data are the first description of a mechanism allowing *Mtb* to respond to host immune activation that may be important in the immunopathogenesis of TB and have use in novel eradication strategies.

## Abstract (web summary)

Mycobacterium tuberculosis senses IFN-γ via the membrane protein MmpL10, which results in stimulation of bacterial respiration and virulence gene expression, worsens infection and paradoxically prevents the formation of isoniazid tolerant persisters.

## Introduction

Tuberculosis (TB) remains a significant public health challenge and is a leading cause of death from a single infectious disease globally. Infection with the causative pathogen, *Mycobacterium tuberculosis* (*Mtb*), initiates a series of highly complex inflammatory events, primarily in the lung, the intricacies of which have yet to fully elucidated^1^. The ancient relationship between the human host and *Mtb* has resulted in the acquisition of various survival mechanisms by *Mtb* that allow it to avoid immune destruction and establish persistent infection^2^. Approximately 90% of infected individuals do not progress to clinical disease and, although the determinants of protective immunity against *Mtb* infection are not fully understood, several indispensable factors have been identified. The importance of adaptive immunity in humans, for example, is indicated by a direct correlation between the loss of CD4 T cells due to HIV infection and increasing risk of developing active TB. In addition, the importance of proinflammatory cytokines such as tumor necrosis factor-α (TNF-α), interleukin-1β (IL-1β), interleukin-12 (IL-12), and interferon-γ (IFN-γ) is demonstrated by the association between genetic deficiencies in their signaling pathways and increased risk of TB disease^3^. IFN-γ is thought to be a particularly important component of the adaptive response to *Mtb*, because activation of macrophages by IFN-γ is required to restrict mycobacterial replication^4,5^.

On the other hand, overexpression of IFN-γ by CD4 T cells causes mice to succumb rapidly to *Mtb*, a process which appears to be limited by T-cell expression of the inhibitory receptor programmed cell death protein (PD-1)^6^. Perhaps the most striking evidence for this comes from the fact that blockade of the PD-1 axis in cancer patients, which re-activates IFN-γ production by T cells, has led to numerous case reports of latent TB reactivation^7^. How inhibition of this pathway causes TB reactivation is, however, not well understood. Intriguingly, several bacterial pathogens have evolved the ability to directly sense and respond to host cytokines to facilitate their survival and transmission. For example, virulent *Escherichia coli* (*E. coli)* accelerates growth in response to IL-1β, but avirulent *E. coli* does not, indicating that detection of host immune signals is an adaptation in pathogenic strains^8^. Similarly, Gram-negative bacteria have been found to interact with TNF-α, leading to an increase in cellular invasion^9^, whilst sensing of human IFN-γ drives *Pseudomonas aeruginosa* (*P. aeruginosa*) towards a virulent phenotype^10^. In light of these observations and others, we hypothesize that *Mtb* may have evolved the ability to directly sense IFN-γ as a countermeasure to host immunity.

## Results

### Host IFN-γ induces a rapid increase in *Mtb* respiration

**Fig. 1:**
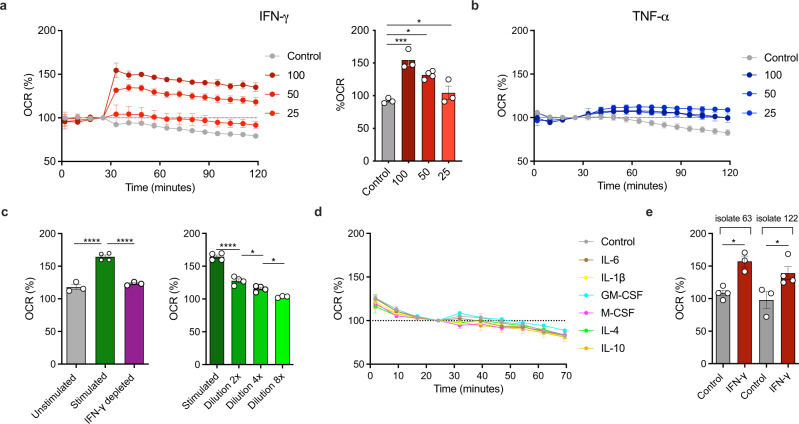
IFN-γ stimulates respiration in *Mtb*. Addition of recombinant human IFN-γ to *Mtb* at indicated concentrations (ng/mL) induces dose-dependent increase in OCR (**a**) whilst no effect was observed with recombinant human TNF-α (**b**). Media derived from stimulated (anti-CD3/CD28 treated) PBMC media, likewise increased OCR, which was abrogated upon IFN-γ depletion (**c**). Dilutions of stimulated media led to a dose-response reduction of the effect. This effect appears to be specific to IFN-γ as several cytokines failed to induce increased respiration (**d**). Clinical *Mtb* isolates also increased OCR in response to 100 ng/mL recombinant human IFN-γ (**e**). Data are shown as mean ± SEM of *n* = 3–4 technical replicates and represent at a minimum two independent experiments. Tukey’s correction multiple-comparison test was used for the statistical analysis. *p < 0.05, ***p < 0.001, ****p < 0.0001.

First, to determine if there was any direct physiological effect of human IFN-γ on *Mtb*, we made use of the Agilent Seahorse XF Analyzer, a highly sensitive analytical platform capable of measuring changes in bacterial respiration^11^. Upon addition of recombinant human IFN-γ, *Mtb* H37Rv rapidly increased oxygen consumption rate (OCR), a proxy for metabolic activity, in a dose-dependent manner (Fig. 1a). By comparison, recombinant human TNF-α had no effect on *Mtb* OCR (Fig. 1b). To rule out the possibility that this observation was due to the use of recombinant human IFN-γ, we stimulated T cells non-specifically for 48 h and then used the culture supernatant to stimulate *Mtb*. This also triggered a rapid increase in *Mtb* OCR, which was abrogated by depleting IFN-γ using a monoclonal antibody (Fig. 1c, left panel), confirming that the effect was specific to IFN-γ. Furthermore, as with recombinant IFN-γ, this effect was dose-dependent (Fig. 1c, right panel). These data also suggest that *Mtb* can sense physiologically relevant concentrations of IFN-γ. No effect on *Mtb* OCR was observed when the recombinant cytokines IL-6, IL-1β, GM-CSF, M-CSF, IL-4 and IL-10 were used (Fig. 1d). We also confirmed that recombinant murine IFN-γ had the same effect on *Mtb* OCR, implying that this phenomenon may be relevant in studies using mouse models of TB (Fig. S1). To further validate this phenotype, we next tested two clinical *Mtb* isolates, derived from human TB subjects recruited from TB clinics in the Durban area and passaged only twice. We confirmed that both strains upregulated the OCR in response to IFN-γ (Fig. 1e). In sum, these data demonstrate that *Mtb* bacilli possess the ability to senses IFN-γ, causing an increase in respiration.

### *Mtb* binds IFN-γ on the bacterial cell surface

**Fig. 2:**
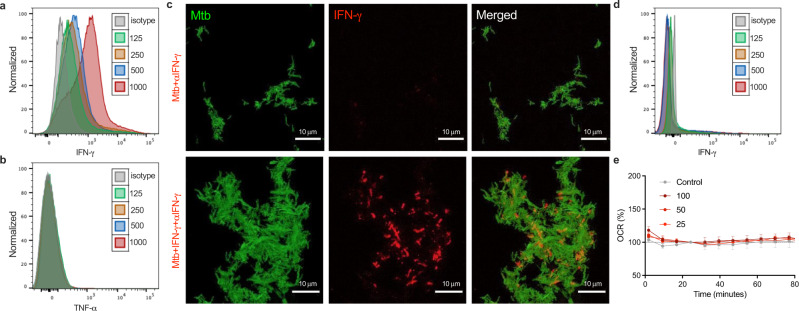
IFN-γ binds to surface of *Mtb* but not BCG. Flow cytometry shows that recombinant human IFN-γ (**a**) but not recombinant human TNF-α (**b**) binds to *Mtb* in a dose-dependent manner at the indicated concentrations (ng/mL). Confocal microscopy confirmed binding of recombinant human IFN-γ to individual *Mtb*-GFP (**c**). Top panel shows control sample; bottom panel shows fully stained sample. In contrast to *Mtb*, BCG does not bind IFN-γ (**d**) nor increase respiration in response to IFN-γ at the indicated concentrations (ng/mL) (**e**). Data are shown as mean ± SEM of *n* = 3–5 technical replicates and represent at a minimum two independent experiments. Tukey’s correction multiple-comparison test was used for the statistical analysis.

Next, we sought to determine how *Mtb* bacilli interact with IFN-γ. The outer membrane of *Mtb* contains various proteins that facilitate transport of molecules, indicating that *Mtb* has evolved mechanisms for binding macromolecules via surface components^12^. To evaluate the interaction between *Mtb* and IFN-γ, we incubated formalin-fixed whole *Mtb* with recombinant IFN-γ followed by staining with a fluorescent anti-IFN-γ antibody. Subsequent flow cytometry analysis indicated that IFN-γ binds to the surface of *Mtb* in a dose dependent manner (Fig. 2a). This result was confirmed by enzyme-linked immunosorbent assay (ELISA) (Fig. S2), and by confocal microscopy (Fig. 2c). In contrast, TNF-α, which failed to induce an increase in OCR, did not bind to *Mtb* using the same experimental method (Fig. 2b). Interestingly, IFN-γ did not bind to the attenuated vaccine strain, Bacillus Calmette–Guérin (BCG) (Fig. 2d). Consistent with the apparent lack of a direct interaction, no change in OCR was detected following addition of IFN-γ to BCG (Fig. 2e). Taken together, these data indicate that the ability of pathogenic mycobacteria to respond to IFN-γ stimulation results from a direct interaction with the cytokine at the cell surface.

### MmpL10 is the binding partner of IFN-γ on the surface of *Mtb*

**Fig. 3:**
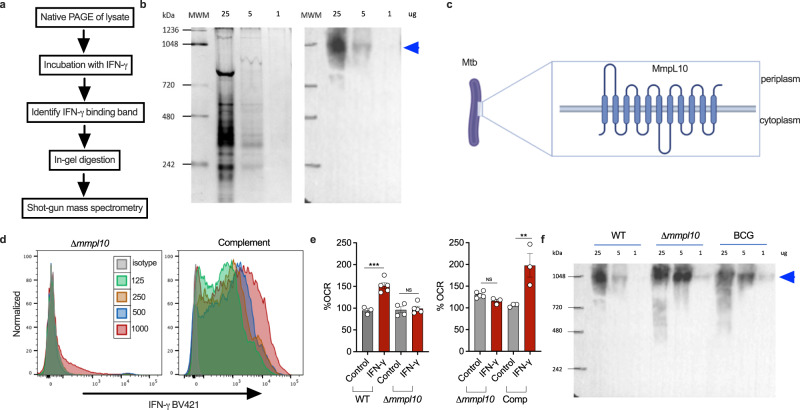
MmpL10 is the binding partner of IFN-γ. A proteomics approach to identify proteins in *Mtb* lysate that bind IFN-γ (**a**). Proteins from native page of *Mtb* lysate (**b**, left panel) were transferred to PVDF membrane. Subsequent staining of the membrane yielded an immunoreactive band to IFN-γ (**c**, right panel). Subsequent analysis using mass spectrometry revealed the band to contain peptides corresponding to MmpL10 (**c**). Binding of IFN-γ to *Δmmpl10* was abolished, while it was restored in the *mmpl10*-complemented strain (**d**). Similarly, the effect on OCR in response to IFN-γ was abolished in *Δmmpl10* and restored in *mmpl10*-complemented strain (**e**). Immunoreactive bands were also observed in lysates of *Δmmpl10* and BCG (**f**). Data are shown as mean ± SEM of *n* = 3–5 technical replicates and represent at a minimum two independent experiments. Tukey’s correction multiple-comparison test was used for the statistical analysis. **p < 0.01, ***p < 0.001.

To further validate these findings, we sought to identify the binding partner of IFN-γ using an unbiased mass spectrometry-based proteomics approach (Fig. 3a). Whole cell lysate of *Mtb* was separated by non-denaturing gel electrophoresis, transferred to polyvinylidene difluoride (PVDF) membrane by Western blotting and incubated with recombinant IFN-γ. The PVDF membranes were then stained with anti-IFN-γ antibody, revealing a distinct IFN-γ binding band (Fig. 3b). The corresponding area was excised from the reference gel and analyzed using LC/MS-MS mass spectrometry. Multiple experiments yielded peptides corresponding to several different *Mtb* proteins (Table S1). Based on the observation that IFN-γ binds on the surface of *Mtb*, we refined the list down to proteins located within the bacterial membrane, leaving only a single characterized candidate, mycobacterium membrane protein large 10 (MmpL10) (Fig. 3c). The same unique peptide of MmpL10 was consistently observed in three independent experiments (Fig. S3), strongly suggesting an interaction between MmpL10 and IFN-γ. To test this putative IFN-γ binding partner, we obtained a *Δmmpl10* transposon mutant of *Mtb*^13^ and assessed binding to IFN-γ as described above. The *Δmmpl10* mutant did not bind IFN-γ (Fig. 3d), and complementation of the *Δmmpl10* mutant with wildtype *mmpl10* (*rv1183*) restored the binding of IFN-γ (Fig. 3e). Crucially, the *Δmmpl10* mutant also did not increase the OCR in response to IFN-γ, but this response was restored in the *mmpl10*-complemented *Mtb* (Fig. 3e). Indeed, overexpression of *mmpl10* in the complemented strain relative to *Mtb* H37Rv, as shown by a higher median fluorescent intensity in the flow cytometric binding experiment, was matched by a greater increase in OCR on addition of IFN-γ (Fig. 3e). To exclude the possibility that non-specific disruption of the bacterial membrane through mutation of a transmembrane protein affected the response of *Mtb* to IFN-γ, we tested the effect of IFN-γ on additional *Δmmpl* mutants available (MmpL-4, 5, 8 and 11), all of which are predicted transmembrane proteins. As expected, all of these *mmpl* mutant strains retained the ability to up regulate OCR in response to IFN-γ (Fig. S4). Having determined that MmpL10 is the binding partner for IFN-γ in *Mtb*, we analyzed its sequence in BCG, which did not bind or respond to IFN-γ. Intriguingly, the *mmpl10* gene in BCG is 100% homologous to that in *Mtb* H37Rv. To explore this further, we tested whole bacterial lysates of both BCG and the *Δmmpl10* mutant used above and found the same distinct IFN-γ binding band in both as observed in *Mtb* H37Rv (Fig. 3f). The *Δmmpl10* transposon mutant used in this experiment contains an insertion at position 2396, corresponding to the transmembrane portion of the 3008-base pair *mmpl10* gene (Table S2). Therefore, it is likely that the portion that binds IFN-γ is retained, explaining why it is still detected in the lysate. However, mutation within the transmembrane domain would be expected to impact the extracellular conformation of MmpL10 preventing the *Δmmpl10* mutant from binding and respond IFN-γ. As the same phenotype is observed for BCG, despite 100% sequence homology, we hypothesize that MmpL10 is differentially localized in BCG.

### Molecular modeling reveals a putative binding site of IFN-γ on MmpL10

**Fig. 4:**
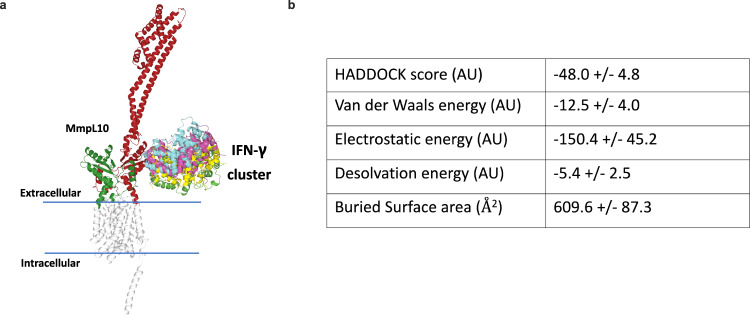
A structural model indicating a putative binding pose for IFN-γ on MmpL10. The first and second extracellular regions of MmpL10 are colored green and red, respectively (**a**). The cluster of potential IFN-γ conformations can be seen contacting the membrane proximal region of the second MmpL10 extracellular region. Scoring metrics were obtained from unguided docking experiments probing the MmpL10-IFN-γ docking cluster generated by HADDOCK, demonstrating reduced energy indicating an interaction (**b**).

In an attempt to understand where IFN-γ may bind to MmpL10, in silico molecular modeling was performed. Unguided docking experiments between IFN-γ and the alphafold model of MmpL10 suggest a potential binding pose in the membrane proximal area of the second MmpL10 extracellular region (Fig. 4a). A singular binding region was predicted, comprised of a cluster of four potential models. This interaction had a negative HADDOCK (High Ambiguity Driven protein-protein DOCKing) score, which is a weighted sum of a variety of energy terms, supporting a favorable interaction (Fig. 4b). Owing to the lack of restraints applied to the docking, further experimental work is required to conclusively determine the IFN-γ binding epitope on MmpL10. Nonetheless, the fact that the predicted binding domain is in the periplasmic portion of MmpL10 is consistent with ability of IFN-γ to bind the bacterial cell surface.

### Stimulation with IFN-γ results in upregulation of virulence genes in *Mtb*

**Fig. 5:**
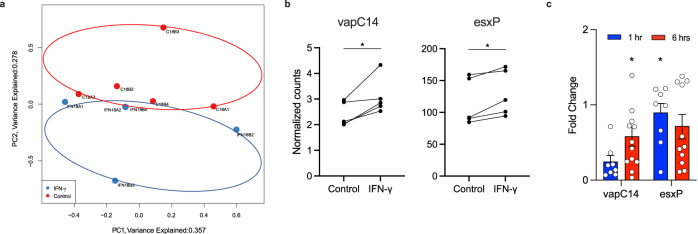
IFN-γ induces transcriptional profile changes in *Mtb*. A distinct transcriptional profile is observed in *Mtb* when exposed to IFN-γ when analyzed by RNAseq and PCA (**a**) and IFN-γ regulates several genes of interest associated with virulence (**b**). This was confirmed by qPCR, which showed significant increase in these genes at 1 h and 6 h post-stimulation (**c**). Gene expression is presented as fold changes normalized to housekeeping gene. Data are shown as mean ± SEM of *n* = 8–12 technical replicates and represent at a minimum two independent experiments. T-test was used for the statistical analysis. *p < 0.05.

Next, to determine whether stimulation with IFN-γ was linked to a transcriptional program in *Mtb*, we incubated multiple separate *Mtb* cultures at mid-log phase growth for 18 h in the presence of the cytokine and sequenced the extracted RNA. Paired-end reads were aligned to the H37Rv reference transcriptome and counts per gene were obtained. Principal component analysis showed a strong batch effect, indicating a high degree of variability between different cultures (Fig. 5a). Despite this, a separation of IFN-γ stimulated and unstimulated bacteria was observed, supportive of the transcriptional reprogramming of *Mtb* in response to IFN-γ, with interquartile range analysis showing no outliers (Fig. S5). Bioinformatic analysis of differentially expressed genes revealed that treatment with IFN-γ significantly regulated several genes (*p* value < 0.001, *q* value < 0.2, Table S3), including upregulation of *vapC14* (rv1953) and *esxP* (rv2347c), encoding putative virulence factors (Fig. 5b). Recently, VapC4, which, like *vapC14* is an RNase toxin, was shown to activate stress survival pathways in *Mtb*^14^. The Esx-1 secretion system is an exporter of virulence factors including the ESAT-6-like protein encoded in *esxP*^15^. Whilst the function of *esxP* is not known, the protein was one of a limited number found to be differentially expressed by *Mtb* H37Rv compared to attenuated strain H37Ra, implying a potentially important role in bacterial virulence^16^. To confirm these results, we conducted additional experiments and measured gene expression of the two putative virulence genes, *vapC14* and *esxP*, by RT-PCR. Using this approach, we confirmed the rapid upregulation of both genes at 1 h and 6 h after stimulation with IFN-γ (Fig. 5c). Collectively, these data demonstrate that virulent *Mtb* has the ability to sense host IFN-γ, via the MmpL10 protein, leading to transcriptional changes that may enhance its virulence and survival.

### IFN-γ accelerates *Mtb* growth in a 3D granuloma model

**Fig. 6:**
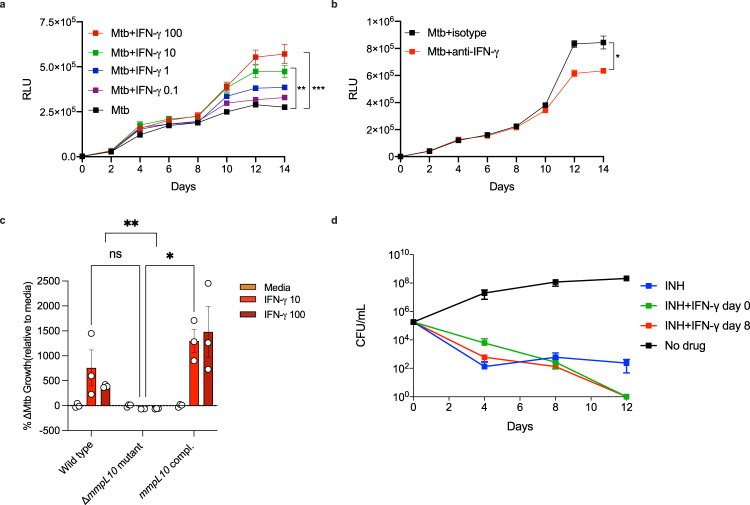
IFN-γ promotes *Mtb* growth via MmpL10. Mtb-infected human PBMCs were encapsulated in 3D microspheres. IFN-γ increases bacterial growth (**a**) measured by relative light units (RLU) in a dose-dependent manner at the concentrations (ng/mL) indicated. In accordance, blockade of IFN-γ reduces bacterial growth (**b**). The effect of IFN-γ at the concentrations indicated (ng/mL) on bacterial growth compared between wildtype *Mtb*, Δ*mmpl10* mutant and *mmpl10*-complement in 3D microspheres, showing no effect of IFN-γ on the Δ*mmpl10* mutant strain (**c**). IFN-γ stimulation enhances INH-mediated killing of Mtb (**d**). Data is shown as ± SEM of *n* = 3 technical replicates and represent at a minimum two independent experiments. CFU data is shown as the percentage of bacterial growth in media containing IFN-γ compared to media. Tukey’s correction multiple-comparison test and Mann–Whitney test was used for the statistical analysis. ns=not significant *p < 0.05, **p < 0.01, ***p < 0.001.

To investigate the potential downstream effects of IFN-γ sensing by *Mtb*, we employed a 3D tissue-like model that mimics several key aspects of the human TB granuloma^17^. Human PBMCs were infected with luminescent *Mtb*, suspended in a matrix consisting of alginate and collagen and finally encapsulated into microspheres. Consistent with earlier experiments using this system, supplementation of recombinant IFN-γ in the culture media increased bacterial growth in a dose-dependent manner, as shown by an increase in luminescent signal (Fig. 6a)^18^. Conversely, depletion of IFN-γ in this model using antibodies significantly reduced bacterial growth by day 14 in culture (Fig. 6b). We next studied the role of MmpL10 in mediating the effect of IFN-γ on bacterial growth, using a conventional colony counting approach as a luminescent *Δmmpl10* mutant strain was not available. In contrast to wildtype *Mtb*, IFN-γ supplementation did not augment CFU count of the *Δmmpl10* mutant (Figs. 6c and S6). Furthermore, this phenotype was rescued in the *mmpl10*-complemented strain, demonstrating that MmpL10 mediates the IFN-γ-induced increase in *Mtb* burden. Interestingly, adding IFN-γ to broth cultures of *Mtb*, in the absence of immune cells, did not lead to a significant increase in growth (Fig. S7). This suggests that the MmpL10-dependent increase in bacterial load induced by IFN-γ is due to augmentation of bacterial fitness and resistance to immune destruction rather than enhanced bacterial growth per se.

### Killing of *Mtb* by isoniazid is enhanced when combined with IFN-γ

Finally, having demonstrated that IFN-γ upregulates bacterial respiration, we tested the effect of IFN-γ stimulation of *Mtb* on drug sensitivity. A major cause of antibiotic treatment failure is thought to be the existence of a population of persister bacteria. These are genetically susceptible organisms that are phenotypically resistant to certain antimicrobial drugs through a myriad of pathways, including a reduction in metabolic activity^19^. The efficacy of the pro-drug isoniazid (INH), for example, is dependent on its conversion by the catalase KatG into its active form. Consequently, sterilizing *Mtb* cultures with INH requires activation of persisters^20^. We therefore hypothesized that IFN-γ stimulation of *Mtb* respiration should prevent the bacilli from entering a metabolically inactive, drug tolerant state. To test this, we added INH to *Mtb* broth cultures with or without the addition of IFN-γ. After 12 days, culturable bacteria were still detectable in the presence of INH alone, indicating the development of persisters as expected (Fig. 6d). In contrast, the addition of IFN-γ, either at the start of the experiment, or on day 8, resulted in culture sterilization by day 12. These findings strongly support the observation that exogenous IFN-γ stimulates *Mtb* metabolism, preventing the formation of drug tolerant persisters and thereby enhancing killing by INH.

## Discussion

Data presented here strongly suggest that *Mtb* possesses the capacity to respond to IFN-γ, thought to be a requisite element in protective TB immunity. We show that virulent *Mtb* binds to IFN-γ and exhibits a dose-dependent increase in metabolic activity, but is unresponsive to other cytokines tested, including TNF-α. This effect was also observed in clinical strains, but not for the attenuated vaccine strain BCG. We identified the transmembrane protein MmpL10 as the putative binding partner for IFN-γ, by showing that the ability to bind and respond to IFN-γ is lost in a *Δmmpl10* mutant strain and is restored by *mmpl10* complementation. This is supported by molecular docking experiments, which identify a putative IFN-γ binding site within the membrane proximal region of MmpL10. Critically, using a 3D granuloma model, we show that IFN-γ promotes *Mtb* growth when MmpL10 is present. RNA sequencing and confirmatory RT-PCR indicates that IFN-γ induces a transcriptional response in *Mtb*, including the upregulation of virulence factors. Finally, the killing of *Mtb* by INH in broth culture was enhanced when combined with IFN-γ, consistent with its ability to induce bacterial respiration. Together, these findings indicate the discovery of a mechanism that allows *Mtb* to sense host IFN-γ, imply an evolutionary adaptation to the host immune response by this highly resilient pathogen.

Adaptation to host immune mediators has been shown in other bacteria, lending biological plausibility to these observations. As a case in point, *P. aeruginosa* has been shown to sense host IFN-γ through the membrane porin OprF, resulting in expression of the virulence factor PA-I^10^. Interestingly, increased IFN-γ does not enhance clearance of *P. aeruginosa*, which could be linked to the subversive action of OprF^21^. As OprF and MmpL10 share little homology in structure or function this appears to be a shared adaption to similar immune pressures and not an example of horizontal gene transfer between bacterial pathogens.

The transmembrane protein family of MmpLs are involved in the establishment of the mycobacterial cell envelope. MmpL10 specifically is thought to be required for the translocation of diacyltrehaloses (DAT) across the plasma membrane, where they are further acylated to generate penta-acyltrehaloses (PAT)^22^. Interestingly, *Mtb* mutants lacking the ability to synthesize DAT were equally infectious as wildtype *Mtb* in mice by either aerosol or intravenous infection, whilst *Δmmpl10* mutant was highly attenuated when given intravenously^23,24^. It is therefore likely that MmpL10 has virulence functions beyond glycolipid transport. The phenomenon of highly conserved proteins involved in metabolic regulation or the cell stress response having a range of additional biological actions which are involved in bacterial virulence, known as “protein moonlighting”, has been described in several bacteria including *Mtb*^25^. Indeed, MmpL3, an integral membrane mycolic acid transporter, was shown to have many binding interactions unrelated to its primary function that allow it to coordinate cell wall deposition during cell septation and elongation^26^. The precise function and mode of action of transmembrane proteins in *Mtb* is complicated by the outer layers of the mycobacterial cell envelope, which separate the plasma membrane from the environment. Although, as discussed, the fact that the putative binding site for IFN-γ is located within the membrane proximal extracellular domain of MmpL10 is consistent with an interaction at the cell surface. In addition, as the transposon insertion site of the mutant used is located in a nearby transmembrane domain, conformational changes of MmpL10 on the cell surface would be expected. Binding of IFN-γ to BCG whole cell lysate is expected as the sequence of MmpL10 is identical and BCG is known to express this protein. However, a comparative analysis of purified membrane fractions of H37Rv and BCG using mass spectrometry revealed several proteins that were significantly more abundant in H37Rv, despite being genetically identical, including MmpL10^27^. Therefore, we hypothesize that either insufficient expression, or differential orientation of MmpL10 in BCG prevent the interaction with IFN-γ at the bacterial cell surface.

How IFN-γ signaling is transduced through MmpL10 is not clear from this study. We identified a number of differentially expressed genes, confirmed by RT-PCR, but this was insufficient to allow bioinformatic identification of the underlying regulatory pathways. An increase in sample numbers, alongside further developments in bioinformatic tools, will be required to link MmpL10 to intracellular *Mtb* signaling pathways. However, *Mtb* is known to sense and response to a number of environmental signals via the two-component signaling system (TCSs)^28^. This highly conserved system among bacteria typically involves a histidine kinase sensor with a periplasmic domain. Triggering of sensor induces an intracellular phosphorylation cascade via specific response regulators, which in turn modify a specific set of genes. The orientation of TCSs sensing molecules, comprising of periplasmic, transmembrane and intracellular domains is consistent with the predicted structure and orientation of MmpL10. In addition, MmpL10 has a threonine phosphorylation site in the C-terminus, which lies in the intracellular juxta-membrane region, and thus might facilitate intracellular signaling^29^. In addition, growing *Mtb* in acetate as a sole carbon source leads to phosphorylation of a single residue at the C-terminus of MmpL11, suggesting that phosphorylation of MmpLs can occur in response to metabolic cues. Further studies are needed to uncover the precise mechanisms involved, but our data indicate that binding of IFN-γ by intact Mtb requires the expression of full length MmpL10 and has physiologically relevant downstream consequences.

Although IFN-γ appears to be indispensable for effective TB immunity, it is also clear that exaggerated Th1 responses, and IFN-γ in particular, can exacerbate disease. Previously, we showed that excessive TNF-α following PD-1 inhibition drives Mtb growth^30^. Lung cavitation, a hallmark of TB disease, is primarily an outcome of unregulated immune responses, adding weight to the notion that *Mtb* thrives in a hyperinflammatory milieu. As highlighted above, a number of studies reported on the development of active TB disease in cancer patients receiving anti-PD-1 therapy, which enhanced IFN-γ production by *Mtb*-specific CD4 T cells^31^. Earlier work also showed that adolescents who exhibited the most intense reactions to tuberculin were more likely to develop TB as adults, many years after the initial tuberculin skin test^32^. Indeed, recent meta-analysis of 34 longitudinal studies reporting the baseline magnitude of the IFN-γ response to *Mtb*, reported that higher levels IFN-γ are consistently associated with a greater risk of active TB^33^. Supporting these clinical observations in humans, PD-1 blockade in macaques caused TB reactivation and PD-1 deficient mice have exaggerated IFN-γ responses and are highly sensitive to *Mtb* infection^34,35^. While there are multiple routes through which excessive IFN-γ production could be detrimental to the host control of *Mtb*, the ability of the pathogen to respond to these conditions, regardless of how they arose, could expedite disease progression. Interestingly, the control of BCG is actually enhanced in PD-1 deficient mice, where it is associated with a significant increase in antigen-specific IFN-γ production by CD4 T cells^36^. Whilst there are many genetic reconfigurations in BCG that contribute to its attenuation, it is possible that its inability to detect and respond to host IFN-γ is a contributing factor.

The long period of human-*Mtb* co-evolution has left a genetic imprint on both parties, which manifest as mechanisms of immune resistance and immune evasion in host and pathogen respectively. Although classified as an intracellular pathogen, *Mtb* can survive and replicate in the extracellular environment^37^. The host pressures that *Mtb* encounters both inside and outside human cells are likely to impact bacterial fitness differently. Further work is necessary to distinguish between these intracellular and extracellular host factors and in what manner they shape the *Mtb* transcriptome. This study suggests sensing host IFN-γ by Mtb improves bacterial fitness and as a result worsens infection. Furthermore, the observation that treatment with IFN-γ leads to culture sterilization by INH indicates that sensing this cytokine drives bacteria out of a persister state. *Mtb* can persist in immune competent hosts as a latent, or quiescent infection for years, which may represent an analogous “persister” state^38^. It is speculated that the ability to develop into a latent infection may have evolved to facilitate spread when human populations were low, requiring the periodic breaking of latency for onward transmission^39^. It is therefore plausible that *Mtb* evolved the ability to sense IFN-γ as a mechanism to reactivate latent *Mtb* infection when the environment was favorable for transmission, for example during a respiratory infection. Importantly, it may be possible to leverage IFN-γ-induced stimulation of *Mtb* respiration to augmenting INH therapy or improve the detection of non-culturable bacteria in clinical samples^40^. In this study, we have demonstrated a hithero unknown effect of IFN-γ on *Mtb*, knowledge of which may help to inform design of future therapies or vaccines to control the TB epidemic.

## Methods

### Bacterial strains and growth conditions

All mycobacterial strains were cultured in Middlebrook 7H9 broth (Difco) (supplemented with 10% OADC, 0.2% glycerol and 0.01% Tyloxapol (Sigma-Aldrich) at 37 °C. *Mtb*-GFP was grown in media containing hygromycin 50 μg/mL. *Mtb Δmmpls* were obtained from the John’s Hopkins University Mutant Library^13^ and grown in media containing kanamycin 25 μg/mL; *Mtb Δmmpl10::*aph complement was grown in media containing 25 μg/ mL kanamycin with 50 µg/mL hygromycin. Bioluminescent *Mtb* H37Rv was grown in media containing 25 μg/mL kanamycin. Live bacteria were used in all Seahorse experiments. Whole bacteria fixed in 4% paraformaldehyde (PFA) were used for Enzyme Linked Immunosorbent Assay (ELISA), confocal microscopy and flow cytometry experiments. Bacterial lysates were prepared for Western blotting. *Mtb* CFU counting was performed by serial dilution in PBS-Tween 80 (0.05%) (Sigma Aldrich) on Middlebrook 7H11 agar with OADC (Difco) unless stated otherwise.

### *Mtb* OCR measurements

OCR measurements were conducted as according to the method of Lamprecht et al.^11^. Briefly, an XFe96 Extracellular Flux Analyser (Seahorse Biosciences) was used to measure the OCR of *Mtb* bacilli. These bacilli were adhered to the bottom of a Cell-Tak coated XF cell culture microplate (Seahorse Biosciences) at 2*10^6^ bacilli per well. Assays were carried out in unbuffered 7H9 media (pH 7.35) without a carbon source. In general, basal OCR was measured for 21 min before the automatic addition of the cytokines (Peprotech) or other stimulants through the drug ports of the sensor cartridge. All OCR figures indicate the point of each addition as a dotted line. OCR data points are representative of the average OCR during 4 min of continuous measurements in the transient microchamber, with the error being calculated from the OCR measurements taken from at least three replicate wells by the Wave Desktop 2.2 software (Seahorse Biosciences).

### IFN-γ binding by ELISA and flow cytometry

Binding of IFN-γ to *Mtb* was assessed by mixing varying concentrations of IFN-γ with whole bacterial cells fixed in 4% PFA and incubating overnight at 4 °C. Bacteria were pelleted and resuspended in 0.1% Tween in PBS before seeding onto microtiter wells for 2 h at 37 °C. After washing, Ultra-LEAF anti-human IFN-γ antibody (Biolegend, clone B27) at 1 μg/mL was added to the wells which were incubated for a further 2 h at room temperature. Anti-mouse HRP at 1:5000 was then added for 1 h at room temperature. TMB solution (Sigma Aldrich) was used as a substrate and 1 M sulfuric acid as stop solution. Optical density was read at 450 nm measured with GloMax Discover microplate reader (Promega). For flow cytometry, after overnight incubation with IFN-γ, bacterial pellets were stained with human anti-IFN-γ Brilliant Violet 421 (Biolegend, clone 4 S.B3) or anti-TNF-α Alexa Fluor 700 (BD, clone MAb11) at 1:20 for 1 h at room temperature. Data was acquired using BD Aria Fusion cytometer and analyzed using FlowJo Software v.10.

### Confocal microscopy

*Mtb*-GFP fixed in 4% PFA was mixed with IFN-γ overnight at 4 °C. Bacteria were stained with anti-human IFN-γ APC (Biolegend, clone 4 S.B3) at 1:25 for 1 h at room temperature. After washing, cells were resuspended in mountant and fixed to the slide. Samples were imaged using an Olympus IX81 microscope and images were exported as lif files and opened in ImageJ.

### Stimulation of PBMCs

Whole blood was collected from healthy donors enrolled in the CUBS study (ethics approved by BREC# BE022/13 at UKZN). Informed consent was obtained from all participants. Peripheral blood mononuclear cells (PBMCs) were isolated from whole blood by centrifugation on Ficoll-Paque (Sigma), resuspended in RPMI 1640 medium (Thermo Fisher) containing 10% Fetal Calf serum, 1% ampicillin and seeded at 2 × 10^5^ cells/well in 96 well plates. Cells were cocultured with Dynabeads Human T-Activator CD3/CD28 (Invitrogen) to stimulate T cells at 37 °C in 5% CO_2_ for 48 h. Supernatants were recovered by centrifugation. IFN-γ was depleted from the supernatant with 5 μg/mL Ultra-LEAF anti-human IFN-γ antibody (Biolegend, clone B27).

### Detection of IFN -γ reactive band on immunoblot

Whole cell lysate was mixed with sample loading buffer without the addition of denaturing or reducing agents. Samples were separated in polyacrylamide gels without addition of SDS after which they were electrotransferred to polyvinylidene difluoride (PVDF) membranes or stained with Coomassie Blue. Following the wash steps and blocking, membranes were incubated with 5 μg of IFN-γ overnight at 4 °C. Membranes were then stained with 5 μg/mL anti-human IFN-γ antibody for 1 h at room temperature followed by anti-mouse HRP at 1:50,000 for 1 h. Membranes were developed with enhanced chemiluminescence reagent (Thermo Fisher) to detect immunoreactive bands. The corresponding bands on the Coomassie stained gel were excised and stored at -80 °C until digestion.

### Protein identification by mass spectrometry

The gel slices were then rinsed with 100 mM ammonium bicarbonate solution and transferred into a sterile Eppendorf^®^LoBind 1.5 ml microcentrifuge tube. A volume of 500 µl of acetonitrile (ACN) was added and the sample was incubated on ice, for 10 min. Following brief centrifugation, the ACN was removed and 100 µl of 10 mM dithiothreitol (DTT) solution was added and incubated at 56 °C for 30 min., removed and cooled to room temperature. Next, 500 µl of ACN was added and incubated on ice for a further 10 min after which the liquid solution was aspirated off, 100 µl of 55 mM iodoacetamide solution was added and the gel sample was incubated at room temperature for 30 min in the dark. Next, 500 µl of ACN was added and the sample was incubated on ice, for 10 min, the liquid solution was then aspirated, and diluted trypsin (Promega, sequence grade) was added to each gel sample to a final concentration of 10 µg per 1 µl of ProteaseMAX^™^ (Promega) 1% trypsin enhancer solution. The sample was then mixed gently and incubated at 4 °C for 2 h. The samples were then incubated in the trypsin/ProteaseMAX^™^ solution at 37 °C overnight. The resulting peptides were extracted by adding 400 µl of 5% formic acid/acetonitrile (1:2, v/v) solution to the sample followed by 15 min incubation at 37 °C on a shaking heating block set at 450 rpm. The samples were then briefly centrifuged, the solution transferred to a sterile Eppendorf^®^LoBind microcentrifuge tube and the sample dried using a SpeedVac concentrator (Labconco, USA) set at 40 °C. The dried, extracted peptides were reconstituted in 50 µl of 5% formic acid solution, and injected for nano-LC-MS/MS analysis. The peptide digests were analyzed using a shotgun analysis on a Thermo Q Exactive Orbitrap mass spectrometer coupled to a Dionex UltiMate 3000 UPLC system. The resultant Thermo RAW files were subjected to analysis using Thermo Proteome Discoverer 2.2, SEQUEST and the *Mtb* H37Rv protein FASTA file obtained from Uniprot. The peptide false discovery rate (FDR) set at <0.01 was used to get confident results.

### Enhancement of isoniazid killing of *Mtb* by IFN-γ

Bacterial cultures were diluted in fresh 7H9 broth containing ADC. Subsequently, mid-log phase *Mtb* H37Rv was diluted to an OD_600_ of 0.01 in 7H9 media (0.2% glycerol, 0.01% Tyloxapol, 10% ADC). These bacilli were aliquoted into inkwells and cultured at 37 °C. Isoniazid was added at a concentration of 1 ug/mL; 100 ng/mL in the case for IFN-γ. 100 µl of the untreated and treated cultures was taken at indicated time points, serially diluted in phosphate buffered saline and plated onto Middlebrook 7H11 agar containing ADC.

### RNA sample preparation

Bacteria were grown to an OD_600_ of 0.6 and then pelleted and treated with RNAprotect Bacteria Reagent (Qiagen) for 15 min at room temperature. Samples were washed with PBS and resuspended in TRIzol (Life Technologies) and stored at -80 °C. Thawed samples were transferred to Lysis Matrix B tubes containing 0.1-mm silica beads (Q-Biogene) and homogenized in a MagnaLyser instrument (Roche) at 7000 rpm for 5 × 60 s with 3 min incubation on ice in between homogenizations. Samples were centrifuged for 1 min at 16,100×*g* at 4 °C, and the supernatant was transferred to a new Eppendorf tube. After phenol-chloroform extraction, the nucleic acids were precipitated with isopropanol, washed with 75% ethanol, air dried for 10 min, and finally resuspended in nuclease-free water. Genomic DNA was removed with RNase-Free DNase Set (Qiagen) and RNA was further purified on-column using RNeasy Mini Kit (Qiagen) and eluted in 50 μL of nuclease-free water. Purity and integrity were verified by Nanodrop (Thermo) and Bioanalyzer (Agilent) respectively.

### RNA sequencing analysis

Paired-end sequence reads were quantified to transcript abundance using Kallisto with bias correction, and 50 bootstrap samples resulting in at least 35.9 million aligned reads per sample. Reads were mapped to *Mtb* H37Rv individual gene sequences database from Mycobrowser release 4. On average, the percentage of aligned reads was 89.4%. The transcript abundance was then summarized to gene level using Sleuth. Raw counts from RNA-sequencing were processed in Bioconductor package EdgeR, variance was estimated, and size factor normalized using Trimmed Mean of M-values (TMM). Genes were filtered using the filtered_p function of the genefilter package. All fit models included a term to model individual variation. For the identification of DEGs a group comparison was applied using experiment batch as covariant. Genes with a FDR-corrected *p*-value < 0.05 were identified as differentially expressed, resulting from a likelihood ratio test using a negative binomial generalized linear model fit. Top genes with a nominal *p* value < 0.01 were also considered for further validation. Pathway enrichment analysis was performed using the cellular overview tool available in the BioCyc database collection based on the *Mtb* H37Rv reference genome.

### Real-time PCR

Following RNA extraction as described above, cDNA was synthesized in 20-µl reaction volumes with the High Capacity cDNA RT kit (Applied Biosystems). The cDNA samples were diluted 1:3 in nuclease-free water, and real-time qPCR was performed with 5-µl reaction volumes containing TaqMan™ Gene Expression Assay (Thermofisher, UK) for the genes esxP (ID# APWC34V), vapC14 (ID# APXGXPT) and 16srna (ID# AP329ZG). Reactions were run on a 7900HT Fast real-time PCR system (Applied Biosystems) with the following program: 2 min at 50 °C, 10 min at 95 °C, and 40 cycles of 15 s at 95 °C and 1 min at 60 °C. All samples were amplified in triplicate, and threshold cycle (CT) values of ≥40 were considered negative. Expression data were normalized to the housekeeping gene, and relative quantifications were carried out by the ΔΔCT method.

### Complementation of *mmpl10* in the mutant strain

The *mmpl10* (*rv1183*) ORF was PCR amplified from *Mtb* genomic DNA using the primers (Thermo, USA) rv1183F(ATGTTCGAAGTGGTCGGCTGTTGGGTCGC), rv1183R(CACGTTAACCCGCCTTCGGCGGCTAAACA) and KOD Xtreme HotStart DNA polymerase (Roche). After PCR clean up, the *mmpl10* PCR product and pMV762 shuttle vector were digested with *Bst*BI and *Hpa*I (Thermo, USA), purified using agarose electrophoresis, and ligated with T4 DNA ligase (NEB) to produce pMV762::*mmpl10*. The pMV762::*mmpl10* complementation vector expressed *mmpl10* under the control the *hsp*_*60*_ promoter and were electroporated using Gene Pulser Xcell (Biorad) into electrocompetent *Δmmpl10 Mtb* transposon mutant. Transformants were selected on 7H10 agar plates containing hygromycin (50 µg/mL).

### 3D cell culture

Microspheres were generated with an electrostatic generator (Nisco, Zurich, Switzerland) to recapitulate the TB granuloma environment^17^. Firstly, PBMC were infected overnight with *Mtb* at a multiplicity of infection of 0.1, in a 75 cm^3^ flask. The next day the cells were detached, pelleted and mixed with 1.5% sterile alginate (Pronova UP MVG alginate, Nova Matrix, Norway) and 1 mg/mL collagen (Advanced BioMatrix, USA) at a final concentration of 5 × 10^6^ cells/ml. The cell-alginate suspension was injected into the bead generator where microspheres were formed in an ionotropic gelling bath of 100 mM CaCl_2_ in HBSS. After washing twice with HBSS with Ca^2+^/Mg^2+^, microspheres were dispensed into eppendorfs and transferred in RPMI 1640 medium (Thermo Fisher) containing 25 μg/mL kanamycin, 1% ampicillin, 10% human AB serum, and incubated at 37 °C, 5% CO_2_. *Mtb* growth within microspheres was monitored longitudinally by luminescence (GloMax 20/20 Luminometer, Promega). In the case of IFN-γ supplementation, the media contained the indicated final concentrations of cytokine. In the IFN-γ blockade experiment, the cell suspension was incubated with 100 μg/mL anti-IFN-γ antibody (Biolegend) for 1 h at 4 °C before addition to the alginate-collagen matrix. For the experiment comparing Mtb wild type, *Δmmpl10* mutant and *mmpl10*-complement, the culture media of the microspheres contained the appropriate antibiotics. CFU counts, microspheres were dissolved in 5 mM EDTA with 1% saponin in HBSS and bacteria were plated onto 7H11 agar. Time points described are days post infection.

### In silico modeling

Unguided docking experiments were performed using the HADDOCK2.4 webserver. The crystal structure of IFN-γ (PDB:1FGY) along with the alphafold model of MmpL10 (AF-P9WJU0-F1) were used as the models for the docking analysis. As no prior restraints were available, random patches were defined during the docking runs, with the number of structures used for rigid body docking set to 10,000, the number of structures for semi-flexible refinement set to 400 and the number of structures for final refinement set to 400. All other parameters were left as default. The proposed binding models were visualized and inspected using PyMOL.

### Statistics and reproducibility

Statistical analyses were performed using GraphPad Prism software v. 9 (San Diego, USA). A calculated *p*-value of  <0.05 was considered to be statistically significant. No statistical methods were used to predetermine sample size. The statistical analyses performed are mentioned in the corresponding figure legends. All experiments presented in the manuscript were reproducible.

### Reporting summary

Further information on research design is available in the Nature Portfolio Reporting Summary linked to this article.

## Supplementary information

Supplementary Information

Description of Additional Supplementary Files

Suppl Data 1

Reporting Summary

## Data Availability

RNAseq dataset will be published on European Nucleotide Archive (ENA), accession number *PRJEB56499*. Uncropped gel and blot images are provided as Supplementary Fig. 8. Source data is provided as Supplementary Data 1.

## Code availability

Programming code will be made available on Github (https://github.com/djgarayb/INFg_Mtb_Ahmed_2022).

## References

[1] O’Garra, A. et al. The immune response in tuberculosis. *Ann. Rev. Immunol.* vol. 31 (2013). 10.1146/annurev-immunol-032712-095939 23516984

[2] Ernst JD (2018). Mechanisms of M. tuberculosis immune evasion as challenges to TB vaccine design. Cell Host Microbe.

[3] Abel, L., El-Baghdadi, J., Bousfiha, A. A., Casanova, J. L. & Schurr, E. Human genetics of tuberculosis: a long and winding road. *Philos. Trans. R. Soc. B Biol. Sci*. **369**, 20130428 (2014). 10.1098/rstb.2013.0428 PMC4024222 24821915

[4] Flynn JAL (1993). An essential role for interferon γ in resistance to mycobacterium tuberculosis infection. J. Exp. Med..

[5] Cooper AM (1993). Disseminated tuberculosis in interferon γ Gene-disrupted mice. J. Exp. Med..

[6] Sakai S (2016). CD4 T cell-derived IFN-γ plays a minimal role in control of pulmonary mycobacterium tuberculosis infection and must be actively repressed by PD-1 to prevent lethal disease. PLoS Pathog..

[7] Langan EA (2020). Immune checkpoint inhibitors and tuberculosis: an old disease in a new context. Lancet Oncol..

[8] Porat R, Clark BD, Wolff SM, Dinarello CA (1991). Enhancement of growth of virulent strains of Escherichia coli by interleukin-1. Science.

[9] Luo, G., Niesel, D. W., Shaban, R. A., Grimm, E. A. & KLIMPELi, G. R. *Tumor Necrosis Factor Alpha Binding to Bacteria: Evidence for a High-Affinity Receptor and Alteration of Bacterial Virulence Properties*. *INFECrION AND IMMUNITY* http://iai.asm.org/ (1993). 10.1128/iai.61.3.830-835.1993 PMC302808 8381771

[10] Wu L (2005). Microbiology: Recognition of host immune activation by Pseudomonas aeruginosa. Science.

[11] Lamprecht DA (2016). Turning the respiratory flexibility of Mycobacterium tuberculosis against itself. Nat. Commun..

[12] Rempel S (2020). A mycobacterial ABC transporter mediates the uptake of hydrophilic compounds. Nature.

[13] Lamichhane G (2003). A postgenomic method for predicting essential genes at subsaturation levels of mutagenesis: application to Mycobacterium tuberculosis. Proc. Natl Acad. Sci. USA.

[14] Barth, V. C. et al. Mycobacterium tuberculosis VapC4 toxin engages small ORFs to initiate an integrated oxidative and copper stress response. *Proc. Natl. Acad. Sci*. *USA* **118**, e2022136118 (2021). 10.1073/pnas.2022136118 PMC8364209 34362841

[15] Ryndak MB, Singh KK, Peng Z, Laal S (2015). Transcriptional profile of mycobacterium tuberculosis replicating in type II alveolar epithelial cells. PLoS ONE.

[16] He, X.-Y., Zhuang, Y.-H., Zhang, X.-G. & Li, G.-L. Comparative proteome analysis of culture supernatant proteins of Mycobacterium tuberculosis H37Rv and H37Ra. 10.1016/S1286-4579(03)00179-5 (2003). 10.1016/s1286-4579(03)00179-5 12919853

[17] Tezera LB (2017). Dissection of the host-pathogen interaction in human tuberculosis using a bioengineered 3-dimensional model. Elife.

[18] Ogongo, P. et al. Tissue-resident-like CD4+ T cells secreting IL-17 control Mycobacterium tuberculosis in the human lung. *J. Clin. Investig*. **131**, e142014 (2021). 10.1172/JCI142014 PMC8121523 33848273

[19] Fisher RA, Gollan B, Helaine S (2017). Persistent bacterial infections and persister cells. Nat. Rev. Microbiol..

[20] Vilchèze C (2017). Enhanced respiration prevents drug tolerance and drug resistance in Mycobacterium tuberculosis. Proc. Natl Acad. Sci. USA.

[21] Murphey ED, Herndon DN, Sherwood ER (2004). Gamma interferon does not enhance clearance of pseudomonas aeruginosa but does amplify a proinflammatory response in a murine model of postseptic immunosuppression. Infect. Immun..

[22] Belardinelli J (2014). Biosynthesis and translocation of unsulfated acyltrehaloses in mycobacterium tuberculosis. J. Biol. Chem..

[23] Rousseau C (2003). Deficiency in mycolipenate- and mycosanoate-derived acyltrehaloses enhances early interactions of Mycobacterium tuberculosis with host cells. Cell. Microbiol..

[24] Lamichhane G, Tyagi S, Bishai WR (2005). Designer arrays for defined mutant analysis to detect genes essential for survival of Mycobacterium tuberculosis in mouse lungs. Infect. Immun..

[25] Henderson B, Martin A (2011). Bacterial virulence in the moonlight: multitasking bacterial moonlighting proteins are virulence determinants in infectious disease. Infect. Immun..

[26] Belardinelli JM (2019). The MmpL3 interactome reveals a complex crosstalk between cell envelope biosynthesis and cell elongation and division in mycobacteria. Sci. Rep. 2019 91.

[27] Gunawardena HP (2013). Comparison of the membrane proteome of virulent Mycobacterium tuberculosis and the attenuated Mycobacterium bovis BCG vaccine strain by label-free quantitative proteomics. J. Proteome Res..

[28] Kundu M (2018). The role of two-component systems in the physiology of Mycobacterium tuberculosis. IUBMB Life.

[29] Prisic S (2010). Extensive phosphorylation with overlapping specificity by Mycobacterium tuberculosis serine/threonine protein kinases. Proc. Natl Acad. Sci. USA.

[30] Tezera, L. et al. Anti-PD-1 immunotherapy leads to tuberculosis reactivation via dysregulation of TNF-α. *Elife* **9**, e52668 (2020). 10.7554/eLife.52668 PMC7058383 32091388

[31] Barber, D. L. et al. Tuberculosis following PD-1 blockade for cancer immunotherapy. *Sci. Transl. Med*. **11**, eaat2702 (2019). 10.1126/scitranslmed.aat2702 PMC7372940 30651320

[32] Comstock GW, Livesay VT, Woolpert SF (1974). The prognosis of a positive tuberculin reaction in childhood and adolescence. Am. J. Epidemiol..

[33] Ledesma, J. R. et al. Interferon-gamma release assay levels and risk of progression to active tuberculosis: a systematic review and dose-response meta-regression analysis. *BMC Infect. Dis*. **21**, 467 (2021). 10.1186/s12879-021-06141-4 PMC8141158 34022827

[34] Kauffman KD (2021). PD-1 blockade exacerbates Mycobacterium tuberculosis infection in rhesus macaques. Sci. Immunol..

[35] Barber DL, Mayer-Barber KD, Feng CG, Sharpe AH, Sher A (2011). CD4 T cells promote rather than control tuberculosis in the absence of PD-1–mediated inhibition. J. Immunol..

[36] Sakai S (2010). PD-1-PD-L1 pathway impairs Th1 immune response in the late stage of infection with Mycobacterium bovis bacillus Calmette-Guérin. Int. Immunol..

[37] Grosset J (2003). Mycobacterium tuberculosis in the extracellular compartment: an underestimated adversary. Antimicrob. Agents Chemother..

[38] Lillebaek T (2002). Molecular evidence of endogenous reactivation of mycobacterium tuberculosis after 33 years of latent infection. J. Infect. Dis..

[39] Blaser, M. J. & Kirschner, D. The equilibria that allow bacterial persistence in human hosts. *Nature*10.1038/nature06198 (2007). 10.1038/nature06198 17943121

[40] Oliver JD (2010). Recent findings on the viable but nonculturable state in pathogenic bacteria. FEMS Microbiol. Rev..

