# Supplementary material for: Mycobacterium tuberculosis senses host Interferon-γ via the membrane protein MmpL10
Source: Commun Biol. 2022 Dec 1;5:1317. doi: 10.1038/s42003-022-04265-0 (PMC9715692; doi:10.1038/s42003-022-04265-0)
Supplement: Supplementary file 1 — Supplementary Information [file 42003_2022_4265_MOESM1_ESM.pdf]

Supplementary Materials for

Mycobacterium tuberculosis senses host Interferon-γ via the membrane protein MmpL10

Mohamed Ahmed[1,2], Jared Mackenzie[1], Liku Tezera[3,5], Robert Krause[1,2], Barry Truebody[1], Diana Garay-Baquero[3], Andres Vallejo[3], Katya Govender[1,2], John Adamson[1], Hayden Fisher[7,8,9], Jonathan W. Essex[4,9], Salah Mansour[3,4], Paul Elkington[3,4], Adrie JC Steyn[1,6], Alasdair Leslie[1,2,5*]

[*]Correspondence to: al.leslie@ahri.org

This PDF file includes:

Figs. S1 to S7
Tables S1 to S3

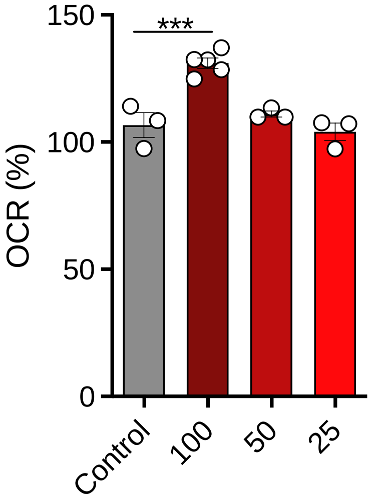

Fig. S1. Murine recombinant IFN-γ increases *Mtb* OCR.

The mouse is the most used animal model used for investigations into basic immunology during Mtb infection. Addition of recombinant murine IFN-γ to *Mtb* at indicated concentrations (ng/mL) induces dose-dependent increase in OCR similar to the effect seen with human IFN-γ. Data are shown as mean ± SEM of n=3-5 technical replicates and represent at a minimum two independent experiments. Tukey's correction multiple-comparison test was used for the statistical analysis. ***$p<0.001$.

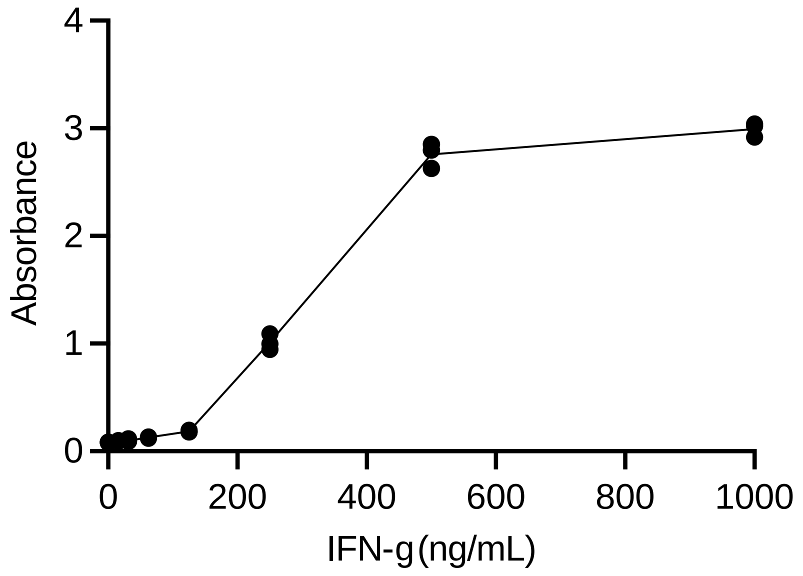

Fig. S2. Binding of IFN-γ to *Mtb*.

IFN-γ binds to formalin-fixed *Mtb* in a dose-dependent manner at indicated concentrations (ng/mL) as measured by ELISA. This assay complements the binding observed by flow cytometry and confocal microscopy. Data are shown as mean ± SEM of n=3 technical replicates and represent at a minimum two independent experiments.

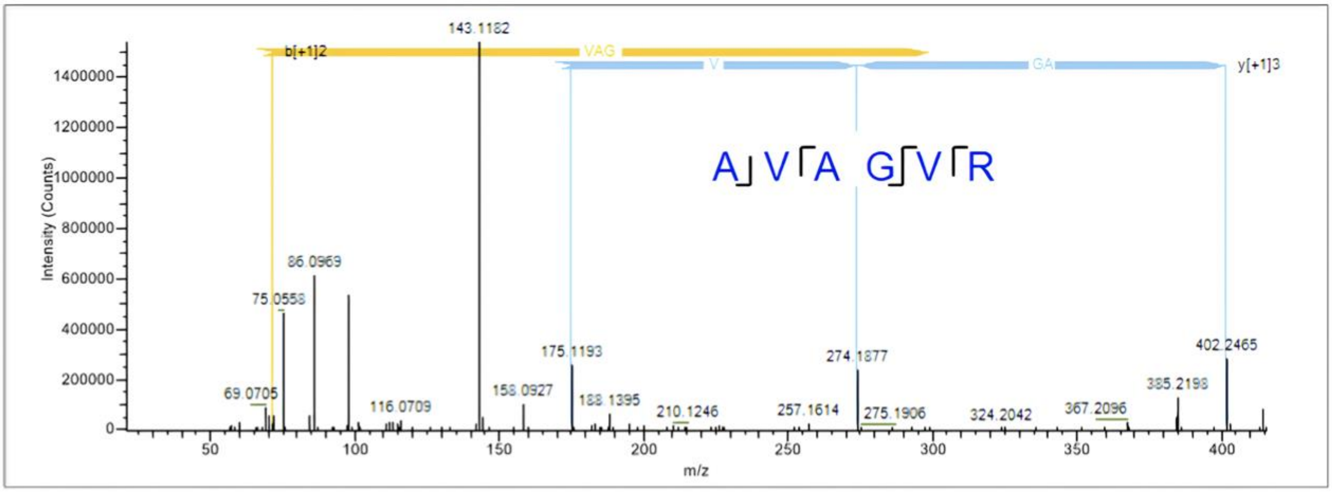

Fig. S3. **Ionic spectrum of unique MmpL10 fragment obtained by HCD fragmentation.**

Unique fragment spectrum to *Mtb* MmpL10 obtained by higher energy collisional dissociation fragmentation.

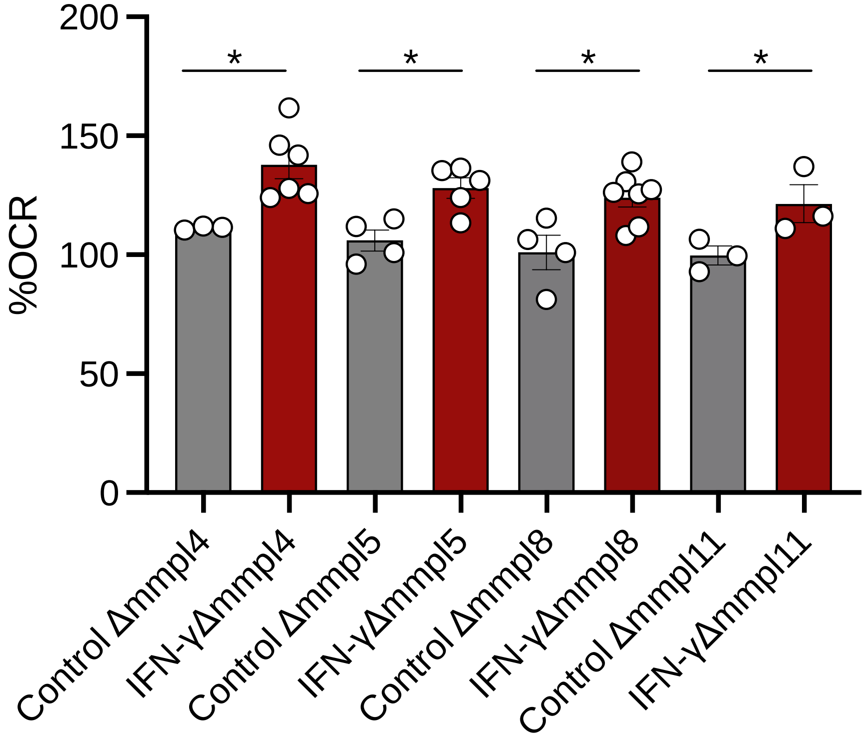

Fig. S4. **IFN-γ increases OCR in several *Mtb Δmmpls*.**

Human recombinant IFN-γ increases OCR in several *Mtb Δmmpls* to confirm this effect is mediated by MmpL10. Data are shown as mean ± SEM of n=3-6 technical replicates and represent at a minimum two independent experiments. Tukey's correction multiple-comparison test was used for the statistical analysis. *p<0.05.

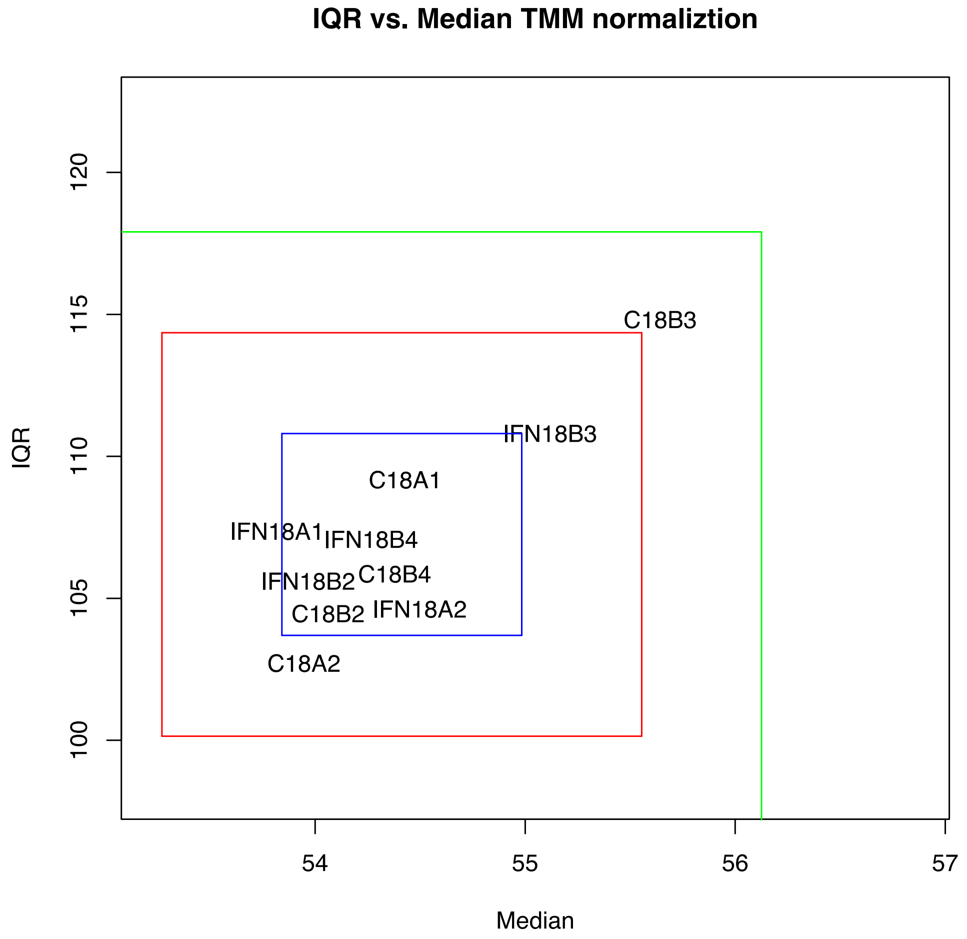

Fig. S5. **Interquartile range analysis of RNA sequencing.**

Control samples denoted with C; IFN-γ-treated samples denoted by IFN. Five replicates per condition. Blue square indicates 1SD, red square 2SD and green square 3SD.

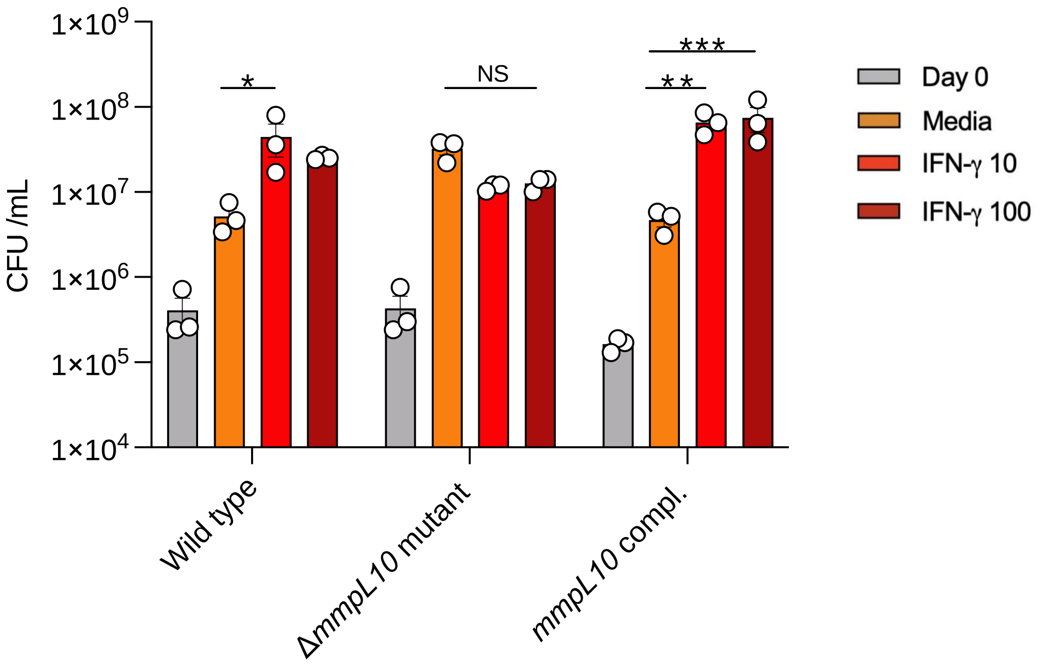

Fig. S6. **Effect of IFN-γ on *Mtb* burden is mediated by MmpL10.**

The role of MmpL10 in IFN-γ-driven *Mtb* infection is investigated by comparing growth of wild-type, Δ*mmpl10* mutant and *mmpl10*-complement. At day 15, IFN-γ supplementation (ng/mL) of the culture media resulted in a statistically significant increased CFU count of *Mtb* wild type but not Δ*mmpl10* mutant. The effect was restored in the *mmpl10*-complement with a higher increase in CFU count compared to wild-type. Data are presented as mean ± SEM of n=3-5 technical replicates and are representative of two experiments done in triplicates. Dunnett's multiple comparison test was used for the statistical analysis. *p<0.05, **p<0.01, ***p<0.001.

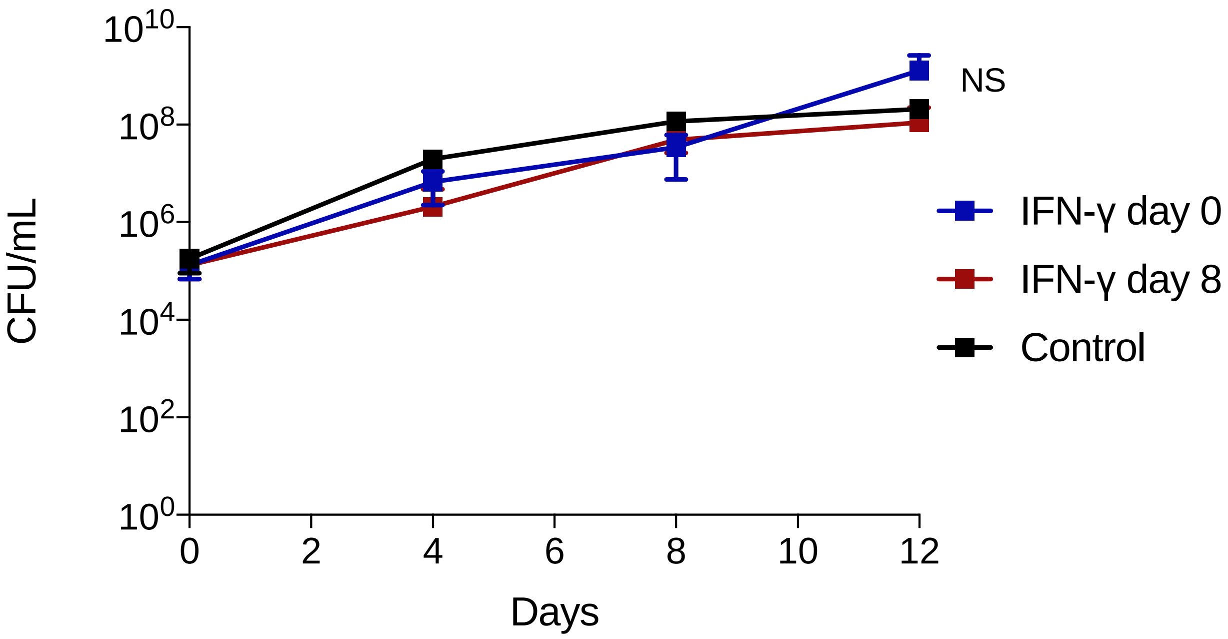

Fig. S7. **IFN-γ does not enhance growth of *Mtb* cultures.**

In contrast to infection of PBMCs, addition of human recombinant IFN-γ to *Mtb* in broth only does not increase bacterial growth. No significant differences in CFU count were observed between after IFN-γ supplementation (100 ng/mL) on day 12. Cytokine was added on day 0 or day 8. Data are presented as mean ± SEM of n=3 technical replicates and are representative of two independent experiments in triplicates. Dunnett's multiple comparison test was used for the statistical analysis. ns = not significant.

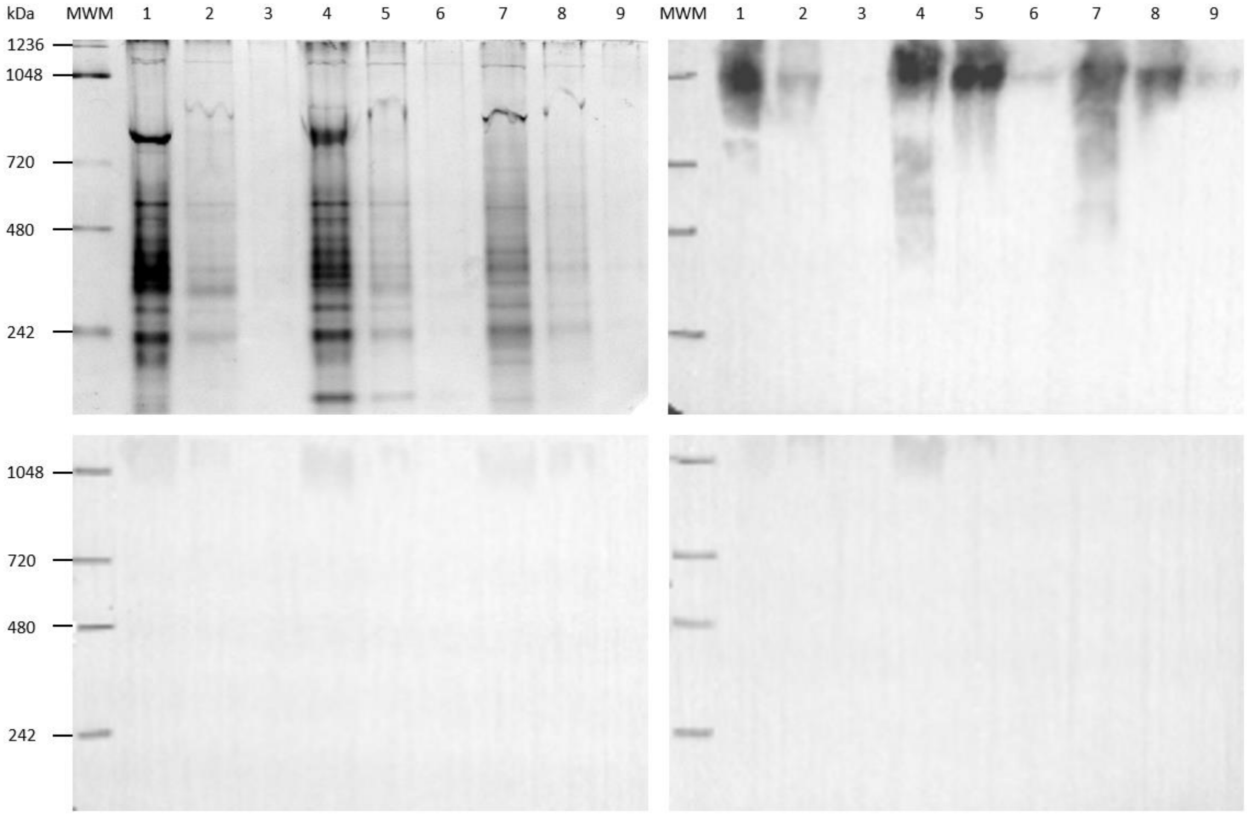

Fig. S8. **Uncropped images of gel and blots.**

Upper left-hand panel: 7.5% native polyacrylamide gel stained with Commassie Blue. Upper right-hand panel: Blot incubated with IFN-γ and anti-IFN-γ antibody. Bottom left-hand panel: Control blot incubated with IFN-γ without anti-IFN-γ antibody. Bottom right-hand panel: Control blot incubated without IFN-γ and with anti-IFN-γ antibody. Lanes 1-3 contain lysate of wildtype *Mtb*. Lanes 4-6 contain lysate of Δ*mmpl10* mutant. Lanes 7-9 contain lysate of BCG. Amount of protein loaded 25 µg, 5 µg and 1 µg per lysate starting at the highest amount. MWM: molecular wight marker. kDa: kilodalton.

Accession	Description	Coverage [%]	# Peptides	# PSMs	# Unique Pepti	# Protein Grour	# AAs	MW [kDa]	calc. pI	Score Sequest HT: Sequest HT
P9WPE7	60 kDa chaperonin 2 OS=Mycobacterium tuberculosis (strain ATCC 25618 / H37Rv) OX=83332 GN=groEL2 PE=1 SV=1	18	6	6	6	1	540	56,7	4,92	2.62
P96400	Probable conserved transmembrane protein OS=Mycobacterium tuberculosis (strain ATCC 25618 / H37Rv) OX=83332 GN=Rv0218 PE=4 SV=1	2	1	1	1	1	442	47,3	10,08	0.00
P9WJU1	Acyltrehalose exporter MmpL10 OS=Mycobacterium tuberculosis (strain ATCC 25618 / H37Rv) OX=83332 GN=mmpL10 PE=1 SV=1	1	1	4	1	1	1002	106,3	8,65	0.00
O05790	Possible phosphatase OS=Mycobacterium tuberculosis (strain ATCC 25618 / H37Rv) OX=83332 GN=Rv3113 PE=4 SV=1	8	1	1	1	1	222	24,8	5,67	0.00
I6Y4V2	Uncharacterized protein OS=Mycobacterium tuberculosis (strain ATCC 25618 / H37Rv) OX=83332 GN=Rv0804 PE=1 SV=1	14	1	1	1	1	209	21,6	12,10	0.00
P9WHR3	Carboxylesterase A OS=Mycobacterium tuberculosis (strain ATCC 25618 / H37Rv) OX=83332 GN=caeA PE=1 SV=1	6	1	1	1	1	520	55,9	6,19	0.00
P9WKH1	(2E,6E)-farnesyl diphosphate synthase OS=Mycobacterium tuberculosis (strain ATCC 25618 / H37Rv) OX=83332 GN=Rv3398c PE=1 SV=1	12	1	1	1	1	359	38,8	6,32	0.00
P9WFQ3	Nucleotide-binding protein Rv1421 OS=Mycobacterium tuberculosis (strain ATCC 25618 / H37Rv) OX=83332 GN=Rv1421 PE=1 SV=1	2	1	1	1	1	301	32,9	6,98	0.00
I6X666	Uncharacterized protein OS=Mycobacterium tuberculosis (strain ATCC 25618 / H37Rv) OX=83332 GN=Rv3076 PE=1 SV=1	5	1	2	1	1	158	17,1	9,95	0.00
P9WN45	1,4-alpha-glucan branching enzyme GlgB OS=Mycobacterium tuberculosis (strain ATCC 25618 / H37Rv) OX=83332 GN=glgB PE=1 SV=1	3	1	1	1	1	731	81,7	5,73	0.00

Table S1. Proteins in immunoreactive membrane bands corresponding to peptide fragments observed by mass spectrometry.

Shotgun mass spectrometry detected several peptides in the immunoreactive band. Only MmpL10 is a characterized membrane-associated protein.

ID	ORF size	POI	ORF description	Rv#	JHU_ID
HG1139	2904	311	MmpL4	Rv0450c	JHU0450c-311
STN1424	2895	1540	MmpL5	Rv0676c	JHU0676c-1540
JO1275	3270	114	MmpL8	Rv3823c	JHU3823c-114
STN0573	3009	2396	MmpL10	Rv1183	JHU1183-2396
NA0234	2901	279	MmpL11	Rv0202c	JHU0202c-279

Table S2. List of *Mtb Δmmpls*.

List of all *Mtb Δmmpls* used in this study taken from JHU transposon mutant library.

Gene	C18A1	IFN18A1	C18A2	IFN18A2	C18B2	IFN18B2	C18B3	IFN18B3	C18B4	IFN18B4	logFC	logCPM	LR	PValue	FDR
Rv3561	2.195.142	198.632	3.008.154	2.551.917	3.000.314	2.501.671	7.962.967	6.115.893	4.266.514	3.819.546	-0.24635	5.224.387	2.908.184	6.94E-08	0.000288
MTB000021	1.504.634	1.773.455	1.203.052	1.337.287	1.167.094	1.271.669	1.003.249	1.316.903	1.780.877	1.932.131	0.203959	7.156.781	2.258.887	2.01E-06	0.002777
Rv1686c	9.040.289	7.875.697	1.647.472	1.370.094	1.581.817	1.238.235	5.571.255	3.837.003	1.668.652	1.565.082	-0.31017	4.340.593	228.777	1.73E-06	0.002777
Rv1687c	1.151.263	9.963.231	2.442.948	1.856.618	2.312.168	1.774.187	8.532.087	6.234.833	2.457.172	2.583.628	-0.28661	4.927.323	1.874.394	1.49E-05	0.015518
Rv2620c	1.548.337	1.745.938	3.447.759	2.259.258	3.273.737	2.251.336	1.218.903	1.052.381	4.132.368	3.347.537	-0.3097	5.484.157	1.595.457	6.49E-05	0.053877
Rv2347c	1.532.075	171.611	8.478.865	9.440.587	9.055.374	1.011.146	9.184.787	1.196.144	1.593.022	1.654.109	0.182111	6.940.971	1.533.315	9.01E-05	0.062364
Rv3559c	1.198.712	1.173.447	1.331.375	1.062.522	1.223.981	1.117.268	2.060.118	1.629.477	1.675.196	1.388.079	-0.22615	3.789.828	1.472.789	0.000124	0.073666
Rv0393	3.789.283	3.185.071	4.224.395	428.737	4.621.408	3.998.642	3.967.373	338.741	3.060.127	2.599.154	-0.17556	5.223.238	1.356.322	0.000231	0.090692
Rv1953	212.272	2.530.344	2.009.623	2.852.033	203.691	2.721.764	2.963.183	4.329.414	2.879.243	3.012.162	0.379653	1.470.671	134.866	0.00024	0.090692
Rv2618	2.816.974	2.941.525	3.747.109	31.391	3.633.407	3.099.787	9.783.208	7.314.807	364.486	3.310.273	-0.20645	5.443.283	1.371.596	0.000213	0.090692
Rv3562	1.892.967	1.698.494	1.948.916	1.638.521	1.789.178	1.634.739	4.465.941	3.480.183	24.539	2.347.623	-0.20024	4.543.133	139.859	0.000184	0.090692
Rv1845c	1.617.263	1.446.724	1.361.938	1.341.201	1.441.802	1.272.509	1.969.341	1.642.799	1.365.023	1.293.987	-0.14118	720.524	1.319.461	0.000281	0.097142
Rv3341	5.728.846	5.544.617	6.518.714	5.845.736	6.538.298	5.307.441	1.298.392	9.308.241	6.877.464	6.744.758	-0.20611	61.584	1.240.264	0.000429	0.136929
Rv3190A	5.886.177	6.373.305	4.354.183	4.524.107	4.398.625	4.736.206	3.226.577	4.291.354	4.780.197	5.412.575	0.170057	5.582.173	1.204.067	0.000521	0.154372
Rv2621c	1.184.478	1.370.182	2.815.147	2.114.978	282.58	2.013.266	7.503.203	5.438.648	2.675.733	233.427	-0.27309	8.242.718	1.129.592	0.000777	0.215012

Table S3. Extended list of differentially expressed *Mtb* genes.

All differentially regulated genes in Mtb after 18 hours IFN-γ incubation. Controls are denoted by C; IFN-γ stimulated samples are denoted by IFN, n=5 per group.
