# Supplementary material for: Mycobacterium tuberculosis senses host Interferon-γ via the membrane protein MmpL10
Source: Commun Biol. 2022 Dec 1;5:1317. doi: 10.1038/s42003-022-04265-0 (PMC9715692; doi:10.1038/s42003-022-04265-0)
Supplement: Supplementary file 4 — Reporting Summary [file 42003_2022_4265_MOESM4_ESM.pdf]

Corresponding author(s): COMMSBIO-21-3115A

Last updated by author(s): Oct 30, 2022

Reporting Summary

Nature Portfolio wishes to improve the reproducibility of the work that we publish. This form provides structure for consistency and transparency in reporting. For further information on Nature Portfolio policies, see our Editorial Policies and the Editorial Policy Checklist.

Statistics

For all statistical analyses, confirm that the following items are present in the figure legend, table legend, main text, or Methods section.

n/a	Confirmed	
☐	☒	The exact sample size (*n*) for each experimental group/condition, given as a discrete number and unit of measurement
☐	☒	A statement on whether measurements were taken from distinct samples or whether the same sample was measured repeatedly
☐	☒	The statistical test(s) used AND whether they are one- or two-sided<br>*Only common tests should be described solely by name; describe more complex techniques in the Methods section.*
☒	☐	A description of all covariates tested
☐	☒	A description of any assumptions or corrections, such as tests of normality and adjustment for multiple comparisons
☒	☐	A full description of the statistical parameters including central tendency (e.g. means) or other basic estimates (e.g. regression coefficient) AND variation (e.g. standard deviation) or associated estimates of uncertainty (e.g. confidence intervals)
☒	☐	For null hypothesis testing, the test statistic (e.g. *F*, *t*, *r*) with confidence intervals, effect sizes, degrees of freedom and *P* value noted<br>*Give P values as exact values whenever suitable.*
☒	☐	For Bayesian analysis, information on the choice of priors and Markov chain Monte Carlo settings
☒	☐	For hierarchical and complex designs, identification of the appropriate level for tests and full reporting of outcomes
☒	☐	Estimates of effect sizes (e.g. Cohen's *d*, Pearson's *r*), indicating how they were calculated

Our web collection on statistics for biologists contains articles on many of the points above.

Software and code

Policy information about availability of computer code

Data collection	Described in methods under "RNA sequencing analysis"
Data analysis	Described in methods under "RNA sequencing analysis"

For manuscripts utilizing custom algorithms or software that are central to the research but not yet described in published literature, software must be made available to editors and reviewers. We strongly encourage code deposition in a community repository (e.g. GitHub). See the Nature Portfolio guidelines for submitting code & software for further information.

Data

Policy information about availability of data

All manuscripts must include a data availability statement. This statement should provide the following information, where applicable:

- Accession codes, unique identifiers, or web links for publicly available datasets
- A description of any restrictions on data availability
- For clinical datasets or third party data, please ensure that the statement adheres to our policy

Data availability statement is included in the manuscript

Field-specific reporting

Please select the one below that is the best fit for your research. If you are not sure, read the appropriate sections before making your selection.

[x] Life sciences [ ] Behavioural & social sciences [ ] Ecological, evolutionary & environmental sciences

For a reference copy of the document with all sections, see nature.com/documents/nr-reporting-summary-flat.pdf

Life sciences study design

All studies must disclose on these points even when the disclosure is negative.

Sample size	No sample size calculation was used. Experiment were repeated with different blood donors
Data exclusions	No data were excluded
Replication	Data is reproducible. All attempts at replication were succesfull
Randomization	Randomization is not relevant to this study. Blood donated by healthy individuals was collected
Blinding	Blinding was not relevant to this study. All participants were healthy

Reporting for specific materials, systems and methods

We require information from authors about some types of materials, experimental systems and methods used in many studies. Here, indicate whether each material, system or method listed is relevant to your study. If you are not sure if a list item applies to your research, read the appropriate section before selecting a response.

Materials & experimental systems

n/a	Involved in the study	
[ ]	[x]	Antibodies
[x]	[ ]	Eukaryotic cell lines
[x]	[ ]	Palaeontology and archaeology
[x]	[ ]	Animals and other organisms
[ ]	[x]	Human research participants
[x]	[ ]	Clinical data
[x]	[ ]	Dual use research of concern

Methods

n/a	Involved in the study	
[x]	[ ]	ChIP-seq
[ ]	[x]	Flow cytometry
[x]	[ ]	MRI-based neuroimaging

Antibodies

Antibodies used	Ultra-LEAF anti-human IFN-□ antibody (Biolegend, clone B27), IFN-□ APC (Biolegend, clone 4S.B3), anti-IFN-□ Brilliant Violet 421 (Biolegend, clone 4S.B3), anti-TNF-□ Alexa Fluor 700 (BD, clone MAb11)
Validation	All information on validation can be found on the manufacturer's website.

Human research participants

Policy information about studies involving human research participants

Population characteristics	Blood donors were healthy individuals not under treatment. Such in-depth information is thus not available.
Recruitment	Healthy blood donors were recruited from local clinics. These individuals were HIV-negative and exhibited no symptoms of TB disease.
Ethics oversight	BREC at University of Kwa-Zulu Natal

Note that full information on the approval of the study protocol must also be provided in the manuscript.

Flow Cytometry

Plots

Confirm that:

☒ The axis labels state the marker and fluorochrome used (e.g. CD4-FITC).

☒ The axis scales are clearly visible. Include numbers along axes only for bottom left plot of group (a 'group' is an analysis of identical markers).

☐ All plots are contour plots with outliers or pseudocolor plots.

☐ A numerical value for number of cells or percentage (with statistics) is provided.

Methodology

Sample preparation	Mycobacteria were fixed by PFA overnight. Cells were washed and resuspended in PBS. Bacteria were stained with IFN-g antibody labelled with BV421 or TNF-a antibody labelled with AF700
Instrument	BD Fusion Aria
Software	Data was anazyled by FlowJo
Cell population abundance	The samples consisted of strictly mycobacteria. Bacteria were cultured in the presence of detergent to ensure single cell suspensions.
Gating strategy	Single bacterial cells were determined by the FSC-A, FSC-H gating. Then intensity of fluorochrome visualized by histograms

☐ Tick this box to confirm that a figure exemplifying the gating strategy is provided in the Supplementary Information.
